# Thyrotoxic periodic paralysis in two sexagenarian men: A case report

**DOI:** 10.1097/MD.0000000000027795

**Published:** 2021-11-24

**Authors:** Ang Lu, Shih-Hua Lin

**Affiliations:** a Department of Medicine, Tri-Service General Hospital, National Defense Medical Center, Taipei, Taiwan; b Division of Nephrology, Department of Medicine, Tri-Service General Hospital, National Defense Medical Center, Taipei, Taiwan.

**Keywords:** elderly, hyperthyroidism, hypokalemia, paralysis, periodic paralysis

## Abstract

**Rationale:**

Thyrotoxic periodic paralysis (TPP) characterized by the triad of muscle paralysis, acute hypokalemia, and the presence of hyperthyroidism is often reported in young adults but rarely reported in age >60 year-old.

**Patient concerns:**

Two sexagenarian males (age 61 and 62) presenting to the emergency department with progressive muscle paralysis for hours. There was symmetrical flaccid paralysis with areflexia of lower extremities. Both of them did not have the obvious precipitating factors and take any drugs.

**Diagnosis:**

Their Wayne scores, as an objective index of symptoms and signs associated with thyrotoxicosis, were <19 (7 and 14, respectively). Their blood pressure stood 162/78 and 170/82 mm Hg, respectively. Their thyroid glands were slightly enlarged. Both of them had severe hypokalemia (1.8 and 2.0 mmol/L). Their presumptive diagnosis of mineralocorticoid excess disorders with severe potassium (K^+^) deficit were made. However, low urine K^+^ excretion and relatively normal blood acid–base status were suggestive of an intracellular shift of K^+^ rather than K^+^ deficit. Hormone studies confirmed hyperthyroidism due to Graves disease.

**Interventions:**

A smaller dose of K^+^ supplementation (only a total of 50 and 70 mmol K^+^, respectively) were prescribed for the patient.

**Outcomes:**

After treatment, their serum K^+^ levels became normal with a full recovery of muscle strength.

**Lessons:**

Our 2 cases highlight the fact that thyrotoxic periodic paralysis must be still kept in mind as the underlying cause of hypokalemia with paralysis and hypertension in elderly patients to avoid missing curable disorders.

## 1 Introduction

Hypokalemic paralysis (HP) is the most common life-threatening emergency among metabolism-induced symmetrical paralysis.^[1,2]^ Based on the pathophysiology of potassium (K^+^) shift and deficit, it can be simply divided into 2 groups: hypokalemic periodic paralysis (HypoPP) due to acute hypokalemia resulting from sudden intracellular K^+^ shifting into cells and non-HypoPP due to chronic profound K^+^ deficiency. With respect to the etiologies, familial periodic paralysis due to voltage-gated sodium (Na^+^; Nav 1.4) and calcium (Cav1.1) channel mutations in the occidentals and nonfamilial thyrotoxic periodic paralysis (TPP) in the orientals are the most common in HypoPP.^[3–5]^ However, gastrointestinal, adrenal, and renal tubular disorders are common for the non-HypoPP subgroups. Unlike non-HypoPP disorders requiring a large K^+^ replacement to replenish the deficit, patients with HypoPP only need minimal K^+^ supplement to avoid rebound hyperkalemia.^[6]^

The first episode of paralysis in TPP usually occurs in younger males. To the best of our knowledge, TPP patients with age more than 60 are extraordinarily rare and may receive a misdiagnosis of non-HypoPP, leading to inappropriate management and the development of life-threatening complication.^[7]^ In this report, we described 2 sexagenarian men presenting with HP and hypertension caused by TPP rather than mineralocorticoid excess disorders with chronic hypokalemia in spite of no obvious thyrotoxic symptoms and precipitating factors. Informed consents were obtained from the patients.

## 2 Case reports

### 2.1 Case 1

A 62-year-old man presented to the emergency department with progressive muscle weakness of both the lower limbs leading to paralysis for 2 to 3 hours. He reported that his blood pressure was higher in the past 2 months without the usage of antihypertensive drugs or diuretics. Moreover, he did not have a family history of muscle paralysis or hyperthyroidism.

His blood pressure, pulse rate, respiratory rate, and temperature were 162/78 mm Hg, 92 beats/min, 18 breaths/min, and 36.7°C, respectively. His thyroid glands were slightly enlarged. Neurologic examination revealed symmetrical areflexia and paralysis in both his lower extremities. The remainder of the physical examination was unremarkable.

Relevant laboratory studies revealed severe hypokalemia (K^+^: 2.0 mmol/L), as shown in Table 1. Electrocardiography revealed high QRS voltage and prominent U waves. A presumptive diagnosis of primary aldosteronism with HP was made. Administration of a high dose of potassium chloride supplement at a rate of 20 mmol/h was planned. However, consultation with a well-experienced nephrologist revealed that his blood acid–base status was relatively normal, and his spot urine examination indicated low urine K^+^ excretion and normal Na^+^ and chloride excretion, suggestive of increased shifting of K^+^ into cells. With a low dose of potassium chloride supplement (<10 mmol/h), he regained his muscle strength at the serum K^+^ 4.2 mmol/L after a total 50 mmol K^+^ supplement. Although his Wayne score was 7 (>19, overt hyperthyroidism), hormone studies indicated a low thyroid-stimulating hormone level (<0.03 μIU/mL), high free thyroxine (2.91 ng/dL), and high thyroid-stimulating hormone receptor antibody but normal plasma renin activity, and aldosterone concentration. His hyperthyroidism and hypertension were controlled with anti-thyroid drug and nonselective β-blocker. Muscle weakness did not recur in the following 1 year.

**Table 1:** Laboratory results.

Item	Normal range	Case 1	Case 2
Sex/age		Male/62	Male/61
Wayne index	<19	12	14
Na^+^	136–145 mmol/L	139	141
K^+^	3.5–5.1 mmol/L	2.0^∗^	1.8^∗^
Cl^−^	98–107 mmol/L	106	107
HCO_3_^−^	22–26 mmol/L	22.5	23
Urea (BUN)	2.9–8.9 mmol/L	4.6	3.9
Creatinine (Cr)	65–110 μmol/L	60	50
TSH	0.25–5.0 μIU/mL	<0.03^∗^	<0.03^∗^
Free T_4_	0.8–2.0 ng/dL	2.91^∗^	2.65^∗^
Urine			
K^+^	mmol/L	13.4	7.7
K^+^/Cr	mmol/mmol	1.4^∗^	1.5^∗^
TTKG		2.7^∗^	2.1^∗^

A value <3 suggests low K^+^ excretion TTKG = (urine K^+^/serum K^+^) ÷ (urine osmolality/serum osmolality) Cl^−^ = chloride, Free T_4_ = free thyroxine, HCO_3_^−^ = bicarbonate, K^+^ = postassium, Na^+^ = sodium, TSH = thyroid stimulating hormone, TTKG = transtubular K^+^ gradient.

∗ Denotes an abnormal value.

### 2.2 Case 2

A 61-year-old man presented with progressive muscle weakness which led to paralysis on waking up in the morning. His personal and family histories were unremarkable. He did not have vomiting or diarrhea, and use diuretics, laxatives, or illicit drugs.

His blood pressure, pulse rate, and temperature were 170/82 mm Hg, 96 beats/min, and 36.1°C, respectively. His muscle power and reflexes were diminished in both lower extremities. The rest of the physical examination was unremarkable. His Wayne score was 14.

His laboratory data revealed severe hypokalemia (1.8 mmol/L) with relatively normal acid–base state and low urine K^+^ excretion as shown in Table 1. Electrocardiography revealed high QRS voltage, T wave inversion, and prominent U waves. Along with 70 mmol K^+^ supplement, his muscle strength regained at serum K^+^ 3.5 mmol/L. Hormone studies confirmed hyperthyroidism due to Graves disease. Oral propylthiouracil 150 mg and propranolol 120 mg daily achieved euthyroid state and normal blood pressure over 3 months without recurrent muscle paralysis.

## 3 Discussion

These 2 men aged more than 60 years exhibited severe HP and newly-found hypertension without the use of diuretics; secondary hypertension with hypokalemia and renal K^+^ wasting (mineralocorticoid excess disorders) were provisionally diagnosed. However, their blood acid–base states were relatively normal along with a low urine K^+^ excretion rate, indicative of acute K^+^ shifting rather than K^+^ deficit. In addition, small doses of K^+^ supplement (approximately 1 mmol/kg) achieved muscle recovery in favor of acute hypokalemia. Despite a lack of overt symptoms and signs of hyperthyroidism, they were proven to have TPP.

Acute symmetrical muscle weakness or paralysis frequently seen in the emergency department can be caused by neuromuscular, metabolic, and psychologic disorders. Among metabolic causes, HP is the most common electrolyte disorder with potentially fatal cardiac arrhythmias and respiratory failure. The measurement of serum K^+^ concentration helps to distinguish HP from non-HP causes, such as Guillian–Barre syndrome and myasthenia gravis. As aforementioned, HP can be simply divided into HypoPP (acute K^+^ shift into cells) and non-HypoPP (chronic massive K^+^ deficit). Due to clinical features indistinguishable between 2 different disorders, the assessment of blood acid–base state and urine K^+^ excretion rate help separate them in addition to careful history taking and detailed physical examination such as blood pressure and thyroid gland enlargement. Patients with non-HypoPP are usually accompanied by abnormal metabolic alkalosis or acidosis, which was not found in these 2 patients. Among the etiologies of HypoPP, TPP remains the most common cause as shown in both patients.^[8]^

The prevalence of TPP is approximately 2% in the Asian population and increasingly reported in western countries due to globalization.^[9,10]^ The pathogenesis of TPP is primarily related to the activation of Na^+^–K^+^-ATPase by elevated thyroid hormone, hyperinsulinemia, and hyperadrenergic state together with reduced K+ efflux in the skeletal muscle, irrespective of the underlying etiologies of hyperthyroidism such as Graves disease, toxic multi-nodular goiter, and excessive ingestion of thyroxin.^[2]^ The affected patients are predominantly young men, with the peak incidence between 20 and 40 years of age, which are compatible with the age-specific distribution of hyperthyroidism.^[8,11]^ One cases series reported 1 TPP patient aged more than 60 years.^[12]^ Together with our 2 patients, TPP in elderly patients albeit rare is still possible.

Compared to young patients with hyperthyroidism, elderly patients with hyperthyroidism usually exhibit more obscure symptoms or signs and even symptomless. However, isolated systolic hypertension and tachyarrhythmia seem to be more common in them. Of note, the prevalence of hypertension is approximately up to 60% to 70% in elderly patients with hyperthyroidism.^[13,14]^ Moreover, elderly thyrotoxic patients with hypertension may have chronic hypokalemia of divert causes such as use of diuretics or laxatives, mineralocorticoid excess state, or concurrent renal tubular disorders. Accordingly, the presence of hyperthyroidism and severe hypokalemia is also not always to be the signature of TPP.^[10]^ In fact, primary hyperaldosteronism, chronic licorice use, and hypertension treated with diuretics have been found to be the most prevalent causes in the elderly with hypertension and severe hypokalemia with neuromuscular weakness and paralysis.^[15]^ The differential diagnosis for elderly people with severe hypokalemia and hypertension is simply shown in Table 2.

**Table 2:** Common causes of profound hypokalemia in the elderly with hypertension.

A.	Increased of K^+^ shifting into cells
1.	Increased insulin activity (eg, conditions with enhanced insulin release or action, drugs with insulin effect)
2.	Increased β_2_-adrenergic activity (eg, stress-induced release of catecholamine, drugs and food with β_2_-adrenergic activity)
B.	Increased of gastrointestinal K^+^ loss
1.	Diseases with gastrointestinal obstruction and diarrhea
2.	Villous adenoma
3.	Laxative abuse
C.	Increased of renal K^+^ loss
1.	Diuretics for hypertension or edema (loop diuretics or thiazide or combined)
2.	Osmotic diuretics (eg, substained hyperglycemia in diabetes mellitus)
3.	Mineralocorticoid excess state (eg, primary hyperaldosteronism, renovascular hypertension, the use of licorice, hydrocortisone, and fludrocortisone)
4.	Acquired renal tubular disorders (eg, acquired Bartter or Gitelman syndrome, autoimmune diseases like Sjogren syndrome, and multiple myeloma)

In conclusion, TPP can occur in elderly patients featuring hypertension and HP without even the obvious precipitating factors or thyrotoxic sign and symptoms. The assessment of the blood acid-base state, urine K^+^ excretion rate, and amount of K^+^ supplement needed to achieve muscle recovery are crucial for the differential diagnosis.

## Author contributions

**Supervision:** Shih-Hua Lin.

**Writing – original draft:** Ang Lu.

**Writing – review & editing:** Shih-Hua Lin.
